# The Development of a Specific and Sensitive LC-MS-Based Method for the Detection and Quantification of Hydroperoxy- and Hydroxydocosahexaenoic Acids as a Tool for Lipidomic Analysis

**DOI:** 10.1371/journal.pone.0077561

**Published:** 2013-10-24

**Authors:** Priscilla B. M. C. Derogis, Florêncio P. Freitas, Anna S. F. Marques, Daniela Cunha, Patricia P. Appolinário, Fernando de Paula, Tiago C. Lourenço, Michael Murgu, Paolo Di Mascio, Marisa H. G. Medeiros, Sayuri Miyamoto

**Affiliations:** 1 Departamento de Bioquímica, Instituto de Química, Universidade de São Paulo, São Paulo, SP, Brazil; 2 Luiz Barssotti Application Laboratory, Waters Technologies from Brazil, São Paulo, SP, Brazil; Max Delbrueck Center for Molecular Medicine, Germany

## Abstract

Docosahexaenoic acid (DHA) is an *n*-3 polyunsaturated fatty acid that is highly enriched in the brain, and the oxidation products of DHA are present or increased during neurodegenerative disease progression. The characterization of the oxidation products of DHA is critical to understanding the roles that these products play in the development of such diseases. In this study, we developed a sensitive and specific analytical tool for the detection and quantification of twelve major DHA hydroperoxide (HpDoHE) and hydroxide (HDoHE) isomers (isomers at positions 4, 5, 7, 8, 10, 11, 13, 14, 16, 17, 19 and 20) in biological systems. In this study, HpDoHE were synthesized by photooxidation, and the corresponding hydroxides were obtained by reduction with NaBH_4_. The isolated isomers were characterized by LC-MS/MS, and unique and specific fragment ions were chosen to construct a selected reaction monitoring (SRM) method for the targeted quantitative analysis of each HpDoHE and HDoHE isomer. The detection limits for the LC-MS/MS-SRM assay were 1−670 pg for HpDoHE and 0.5−8.5 pg for HDoHE injected onto a column. Using this method, it was possible to detect the basal levels of HDoHE isomers in both rat plasma and brain samples. Therefore, the developed LC-MS/MS-SRM can be used as an important tool to identify and quantify the hydro(pero)xy derivatives of DHA in biological system and may be helpful for the oxidative lipidomic studies.

## Introduction

Docosahexaenoic acid [DHA, 22:6 n-3] is an *n*-3 fatty acid that is highly enriched in the brain. DHA is particularly enriched in synaptosomal membranes and synaptic vesicles, and this enrichment suggests a role for this fatty acid in the central nervous system [1]. DHA is critical for normal brain function, and changes in the quantity and/or oxidation of DHA are associated with neurodegenerative diseases [2-5].

**Figure 1:**
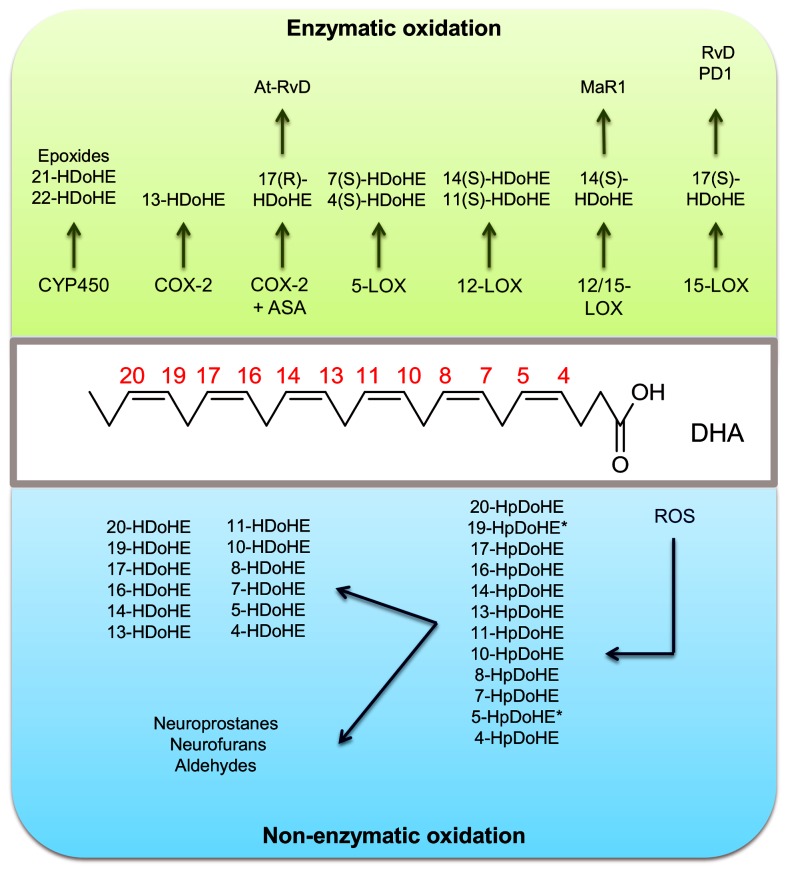
Generation of hydroperoxy, hydroxy and epoxy derivatives of DHA by enzymatic and non-enzymatic mechanisms. *19- and 5-HpDoHE and HDoHE are specifically formed by singlet oxygen oxidation.

DHA has six double bonds and is therefore highly susceptible to oxidation. In biological systems, DHA can be oxidized in two ways: (i) enzymatically via cyclooxygenases (COX) [6,7], lipoxygenases (LOX)[8] or cytochrome P450 (CYP450) [9]; and (ii) non-enzymatically, through reactions with reactive oxygen species (ROS)[10] and transition metal ions [11]. Both oxidation mechanisms produce a large variety of oxidative metabolites, which include a series of hydroperoxide and hydroxide positional isomers (see Figure 1).

Enzymatically, COX-2 converts DHA to 13-hydroxy-DHA (13-HDoHE) [7]. In the presence of aspirin (acetylsalicylic acid, ASA), acetylated COX-2 produces a 17(R)-hydroperoxy-DHA (17(R)-HpDoHE) intermediate, which is reduced to 17(R)-HDoHE and then converted to one of the aspirin-triggered D series resolvins (At-RvD) [7,12]. DHA is also a substrate for LOX enzymes. The 15-LOX converts DHA to 17(S)-HpDoHE and 17S-HDoHE, which can be subsequently converted to D series resolvins (RvD) and protectin/neuroprotectin D1 (PD1) [13]. The 12- and 12/15-LOX convert DHA to 14(S)-H(p)DoHE, which undergoes 13(14)-epoxidation to form maresin 1 (MaR1) [14]. Alternatively, 12-LOX can also generate 11(S)-HDoHE [15] and 5-LOX converts DHA into 2 isomers: 4*S*-HDoHE, and 7(S)-HDoHE; the former is the major isomer [6,16]. The cytochrome P450 (CYP450) enzymes also catalyze the production of two HDoHE (21- and 22-HDoHE) and several epoxides [17]. 

DHA is also oxidized non-enzymatically by ROS. The oxidation mechanism can involve radical or non-radical species, such as singlet molecular oxygen. This excited species can be produced under inflammatory conditions by reactions that involve biological peroxides [18-22]. Both mechanisms lead to the production of HpDoHE as the primary products. Free radical-mediated oxidation of DHA generates ten positional isomers of HpDoHE (20-, 17-, 16-, 14-, 13-, 11-, 10-, 8-, 7- and 4-HpDoHE). In contrast, singlet oxygen-mediated oxidation generates twelve positional isomers, including the same ten isomers produced by radical-induced oxidation and the 5- and 19-HpDoHE isomers. Additionally, the non-enzymatic oxidation of DHA is also reported to generate a series of secondary products, including neuroprostanes, neurofurans and aldehydes (neuroketals and short-chain aldehydes) [23-27]. 

Several lines of evidence indicate that lipid hydro(pero)xides are increase under pathological conditions [28-30]. For this reason, particular attention has been focused on the study of the formation and pathophysiological role of lipid hydro(pero)xides. For example, recent studies on hydroperoxides and other oxy-derivatives of DHA (e.g., resolvins and neuroprotectins) have enhanced our knowledge of the resolution phase of inflammation and of the roles of ASA and *n*-3 in this process [13]. In this context, the identification and structural characterization of HpDoHE and HDoHE formed by enzymatic and/or non-enzymatic mechanisms are critical to reveal new enzymes, products and biological activities [31]. Thus, the aim of this study was to develop a specific and quantitative analytical method suitable for both HpDoHE and HDoHE positional isomers.

Liquid chromatography coupled to tandem mass spectrometry (LC-MS/MS) with the selected reaction monitoring (SRM) method has been widely used for the identification and quantitation of lipid oxidation products. In this study, we present a detailed description of the standardization of a method for qualitative and quantitative LC-MS/MS-SRM analysis of the twelve different positional isomers of both HpDoHE and HDoHE as an additional tool for use in lipidomic studies aiming to dectect DHA oxidation products in biological samples.

## Experimental Section

### Chemicals

4Z,7*Z*,10*Z*,13*Z*,16*Z*,9*Z*-docosahexaenoic acid (DHA), 5*S*-hydroxy-6E,8Z,11Z,14Z-eicosatetraenoic-5,6,8,9,11,12,14,15-*d*8 acid (5(S)-HETE-d8) and 12*S*-hydroxy-5Z,8Z,10E,14Z-eicosatetraenoic-5,6,8,9,11,12,14,15-*d*8 acid (12(S)-HETE-d8) were purchased from Cayman Chemicals Corp. (Ann Arbor, MI). HPLC-grade methanol, acetonitrile, isopropanol, hexane, chloroform, as well as standard grade ammonium hydroxide, phosphoric acid and formic acid were obtained from JT Baker (Avantor Performance Materials, Mexico). Potassium hydroxide (KOH), hydrochloric acid (HCl), 2,6-di-tert-butyl-p-cresol (BHT), deferoxamine mesylate salt, Chelex 100 sodium form and diethylenetriaminepentaacetic acid were purchased from Sigma-Aldrich Inc. (St. Louis, MO). Ketamine hydrochloride and xylazine was obtained from Vet Brands (Sespo Ind. e Com., Brazil). All aqueous solutions were prepared with ultrapure water purified by a Direct-Q3 system (Merck Millipore, Germany) and treated with Chelex 100 before use. Millex Filter Units (0.22 μm) were purchased from Merck Milipore, Germany.

### Preparation of HpDoHE and HDOHE standards

 HpDoHE was synthesized by photooxidation of DHA under an atmosphere saturated with O_2_, and methylene blue was used as the photosensitizer, as previously described (see supporting information method S1 and scheme S1) [21]. The conversion of HpDoHE to HDoHE was performed as described by Terao et al.[32] (see supporting information method S2). The HpDoHE were analyzed and then purified with a Prominence HPLC system (Shimadzu, Tokyo, Japan) equipped with two types of columns: a reverse phase, C18 semi-preparative column (Luna C18-2 100Å, 250 x 10 mm, 5 μm, Phenomenex Inc., Torrance, CA), eluted with a mobile phase of acetonitrile:water:formic acid (70:30:0.005, v/v/v) and water at 4.7 mL/min; and a normal phase, silica semi-preparative column (Luna Silica 100 Å, 250 x 10 mm, 5 μm, Phenomenex Inc., Torrance, CA), with an isocratic mobile phase of hexane:isopropanol:water (99:1:0.1, v/v/v) at 10 mL/min. The PDA detector was set to scan from 200 to 500 nm, and the hydroperoxides were monitored at 205 (all isomers) and 235 nm (isomers with dienes conjugates) (supporting information scheme S1). The fractions containing the isolated isomers were dried with a rotary evaporator, and the residue was solubilized in methanol and stored at -80 °C. An aliquot of each HpDoHE and HDoHE isomer was checked by HPLC- PDA and UV absorbance at 235 nm. HpDoHE concentration was also confirmed by iodometry [33]. 

### Chromatographic standardization

The chromatographic method was developed by a screening study using an automated and integrated system consisting of Fusion Method Development software (S-Matrix Corp., Eureka, CA), Empower 3 chromatography data software (Waters Corp., Milford, MA) and an UHPLC system (Acquity UPLC HClass, Waters Corp., Milford, MA). The screening and optimization study of the chromatographic method was done considering the following parameters: number of peaks, resolution greater than 0.80 and tailing smaller than 2.0. The screening study was performed with four different reversed phase UPLC columns (50 x 2.1 mm, 1.7 µm) the HSS T3, HSS PFP, BEH C8 and CSH Phenyl hexil. Three pH values were evaluated: phosphoric acid 0.1 % (pH 2.5), phosphoric acid 0.05 % (pH 3.5) and ammonium hidroxide 0.1 % (pH 10). Acetonitrile and methanol were used as organic modifier. Gradients between 80 - 100 % of the organic solvent was performed from 5 to 10 minutes and three temperatures were also evaluated 25, 35 and 40 °C. The optimized chromatographic condition consisted of a BEH C8 column (100 x 2.1 mm, 1,7 µm) eluted with a gradient solvent system of A, 0.1 % ammonium hydroxide in water, pH 10; and B, 18 % methanol in acetonitrile at 0.5 mL/min. Elution was started with 30 % B, was held for 1.55 min, was followed by a gradient step to 69% B over 15 min; then, the percentage of B was maintained at 95 % for 2 min and was restored to 30 % for 4 min to allow equilibration. Column temperature was set at 40 °C.

### MS/MS analysis of HpDoHE and HDoHE

The MS/MS fragmentation pattern for each HpDoHE and HDoHE isomer was initially analyzed with a Quattro II triple quadrupole mass spectrometer (Micromass, Manchester, UK) and API 4000 QTrap (Applied Biosystems Inc., Foster City, CA). In this preliminary step, each isolated isomer was identified through a comparison of the obtained fragment ions with the theoretical fragments (Tables S1 and S2 in the supporting information). The final fragmentation study and the establishment of the quantitative method were conducted with an UHPLC system (Acquity UPLC) coupled to a triple-quadrupole mass spectrometer (XEVO TQ-S, Waters Corp., Milford, MA). Based on the MS/MS spectra, the most intense and/or specific fragment ions were selected for the SRM method. The MS and MS/MS analyses were conducted in ESI negative mode. The source temperature was set to 150 °C, the desolvation temperature was 550 °C, and the capillary voltage was set to 3 kV. The dwell time was set automatically as 9 msec. The collision energy and cone voltage were optimized for each compound (see Tables 1 and 2) by the Intellistart tool from MassLynx software (Waters Corp., Milford, MA). Minor adjustments on collision energies were also performed manually for some of the analytes having poor fragment ion intensities. In both cases, the optimization was performed by direct infusion of the isolated standards. The cone energy and collision energy were chosen as the energies which generate the strongest precursor signal and the greatest intensity for the chosen fragment, respectively. Two SRM methods, one for HpDoHE and the other for HDoHE, were created separately to ensure maximum sensitivity in the detection and quantification of the isomers. Peak identification and quantification were performed with TargetLynx software (Waters Corp., Milford, MA). The qualitative SRM was set as target trace and the quantitative SRM was set as quantification trace. The analyte was only quantified in the presence of both transitions.

**Table 1:** Optimized mass conditions for LC-MS/MS method for HpDoHE.

**Compound**	**SRM (*m/z*)**		**CV (V)**	**CE (V)**	**RT (min)**	LOD (pg)*	***r*^2^**	**Calibrated range (ng/µL)**
20-HpDoHE **(2**)	359.10 → 71.10	Quantitative	33	14	6.79	471	0.998	0.25-10
	341.10 → 71.10	Qualitative	33	14				
19-HpDoHE **(1**)	341.30 → 83.10	Quantitative	41	19	6.66	23	0.992	0.01-1
	359.30 → 83.10	Qualitative	41	19				
17-HpDoHE **(4**)	341.30 → 111.20	Quantitative	30	12	7.03	1	0.991	0.01-1
	359.30 → 111.20	Qualitative	30	12				
16-HpDoHE **(3**)	341.20 → 233.20	Quantitative	31	11	7.11	49	0.99	0.01-1
	359.20 → 233.20	Qualitative	31	11				
14-HpDoHE **(6**)	341.10 → 151.10	Quantitative	29	11	7.28	29	0.991	0.01-1
	359.10 → 151.10	Qualitative	29	11				
13-HpDoHE **(5**)	341.10 → 121.10	Quantitative	32	16	7.22	1	0.996	0.01-1
	359.10 → 121.10	Qualitative	32	16				
11-HpDoHE **(7**)	341.10 → 243.20	Quantitative	29	11	7.39	76	0.998	0.01-1
	359.10 → 243.20	Qualitative	29	11				
10-HpDoHE **(7**)	341.10 → 188.20	Quantitative	31	12	7.39	671	0.983	0.01-1
	359.10 → 188.20	Qualitative	31	12				
8-HpDoHE **(8**)	359.30 → 108.30	Quantitative	30	16	7.83	116	0.995	0.01-1
	341.30 → 113.10	Qualitative	31	9				
7-HpDoHE **(8**)	341.10 → 201.20	Quantitative	25	13	7.80	96	0.997	0.01-1
	359.10 → 201.20	Qualitative	25	12				
5-HpDoHE **(9**)	359.20 → 281.30	Quantitative	32	10	8.73	162	0.981	0.01-1
	359.20 → 147.20	Qualitative	32	8				
4-HpDoHE **(10**)	341.30 → 115.10	Quantitative	22	15	8.84	502	0.999	0.01-1
	359.20 → 115.10	Qualitative	22	15				
5(S)-HETE-d8	327.20 → 116.10		22	14	7.25			
12(S)-HETE-d8	327.20 → 184.20		30	14	6.13			

**Figure 2:**
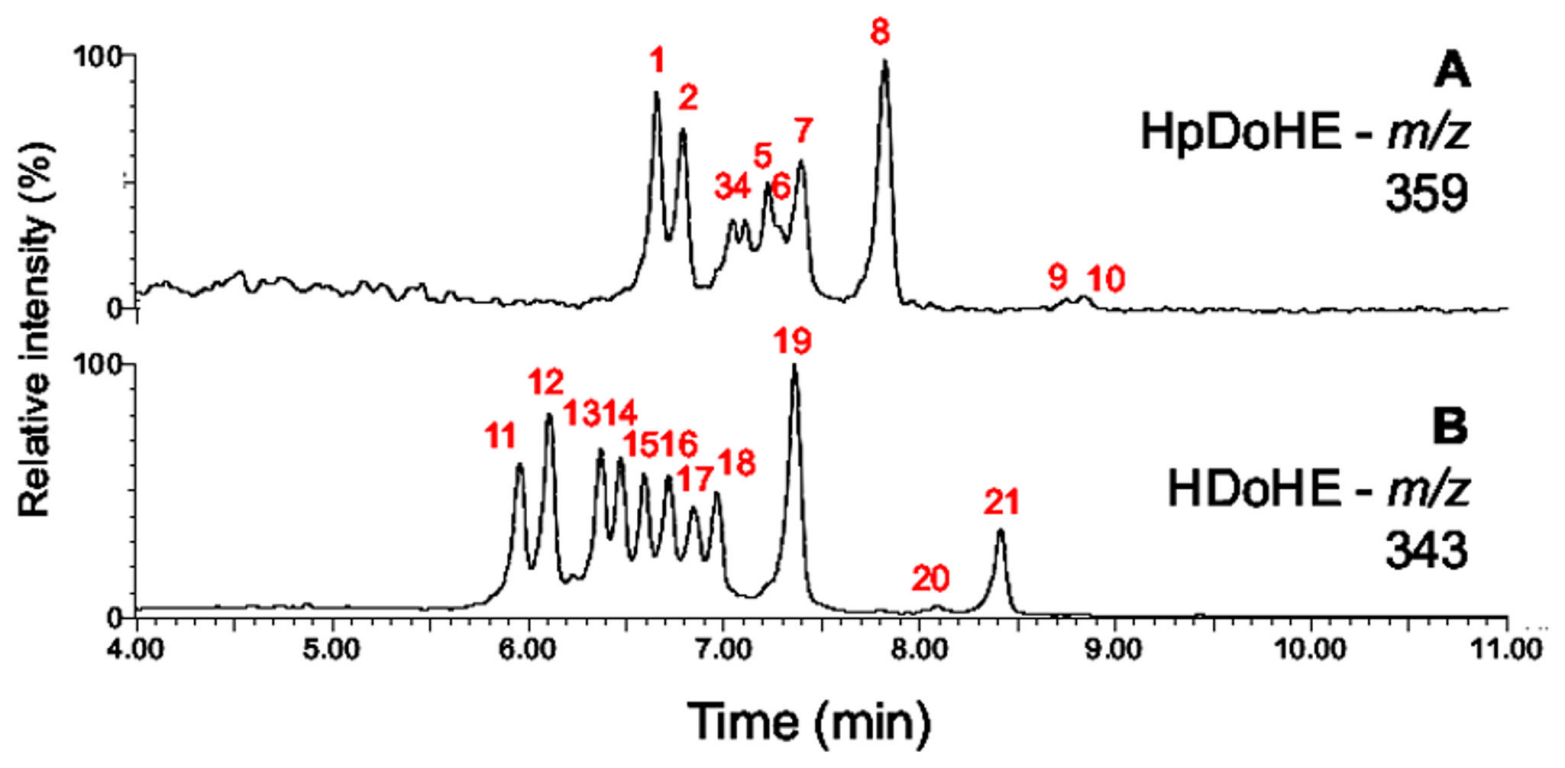
Analysis of a mixture of HpDoHE and HDOHE isomers by UPLC-MS. The HpDoHE and HDOHE isomers were detected through the selection of *m/z* at 359 (A) and 343 (B), respectively. The identities of the peaks numbered in red are shown in Tables 1 and 2 and Figure 5.

Numbers in bold correspond to the peak visualized in the LC-MS analysis (Figure 2). * on column

Optimized mass transitions (*m/z*), cone voltage (CV), collision energy (CE), retention time (RT), limit of detection (LOD) and linearity (*r* ^*2*^).

**Table 2:** Optimized mass conditions for LC-MS/MS method for HDoHE.

**Compound**	**SRM (*m/z*)**		**CV (V)**	**CE (V)**	**RT (min)**	LOD (pg)*	***r*^2^**	**Calibrated range (pg/µL)**
20-HDoHE **(12**)	343.10 → 241.10	Quantitative	36	15	5.95	2.71	0.998	0.62-100
	343.10 → 285.10	Qualitative	36	13				
19-HDoHE **(11**)	343.10 → 229.10	Quantitative	32	15	6.10	5.02	0.999	0.62-100
	343.10 → 273.30	Qualitative	32	14				
17-HDoHE **(14**)	343.10 → 201.10	Quantitative	27	14	6.46	3.89	0.997	1.25-100
	343.10 → 245.30	Qualitative	27	14				
16-HDoHE **(13**)	343.10 → 233.20	Quantitative	26	13	6.36	1.27	0.996	0.62-100
	343.10 → 261.10	Qualitative	26	13				
14-HDoHE **(16**)	343.10 → 161.20	Quantitative	30	12	6.71	2.60	0.996	0.25-100
	343.10 → 205.10	Qualitative	30	15				
13-HDoHE **(15**)	343.10 → 193.20	Quantitative	30	12	6.58	1.16	0.997	0.25-100
	343.10 → 221.10	Qualitative	30	13				
11-HDoHE **(18**)	343.10 → 149.20	Quantitative	28	13	6.95	5.30	0.993	0.25-100
	343.10 → 165.10	Qualitative	28	13				
10-HDoHE **(17**)	343.10 → 153.10	Quantitative	28	13	6.84	2.07	0.998	0.62-100
	343.10 → 181.10	Qualitative	28	15				
8-HDoHE **(19**)	343.10 → 189.20	Quantitative	32	12	7.35	1.63	0.997	0.25-100
	343.10 → 113.10	Qualitative	32	13				
7-HDoHE **(19**)	343.10 → 141.10	Quantitative	31	15	7.36	0.46	0.994	0.25-100
	343.10 → 109.10	Qualitative	31	13				
5-HDoHE **(20**)	343.20 → 85.10	Quantitative	26	10	8.07	8.48	0.994	1.25-100
	343.20 → 93.10	Qualitative	26	10				
4-HDoHE **(21**)	343.10 → 101.10	Quantitative	29	13	8.40	0.50	0.994	0.25-100
	343.10 → 115.10	Qualitative	29	16				
5(S)-HETE-d8	327.20 → 116.10		22	14	7.25			
12(S)-HETE-d8	327.20 → 184.20		30	14	6.13			

Numbers in bold correspond to the peak visualized in the LC-MS analysis (Figure 2). * on column

Optimized mass transitions (*m/z*), cone voltage (CV), collision energy (CE), retention time (RT), limit of detection (LOD) and linearity (*r* ^2^).

### Calibration and validation experiments

The method validation was performed as in-house 3-day protocol to determine linearity, LOD and LOQ, inter- and intra-day variation, and recovery for all compounds. Stock standard solutions of all the HpDoHE and HDoHE isomers were prepared in acetonitrile:methanol:H_2_O (52:18:30, v/v/v) and stored in amber vials at -80 °C. These stock solutions were diluted to prepare the solutions for the calibration curves. The following concentrations were used for the calibration curve: 10, 4, 2, 1, 0.5, 0.25, 0.1, 0.05, 0.025 and 0.01 ng/µL for HpDoHE: and, 4, 2, 1, 0.1, 0.05, 0.025, 0.01, 0.0050, 0.0025, 0.0012, 0.0006 and 0.0003 ng/µL for HDoHE. Isotopically labeled internal standards were prepared in 100 µL of acetonitrile:methanol:H_2_O (52:18:30, v/v/v). For quantitative analysis, 10 µL of 5(S)-HETE-d8 (12.5 ng/µL) and 12(S)-HETE-d8 (12.5 ng/µL) were added to the samples. The injection volume was 10 µL. The calibration curves were constructed by plotting the ratio of the peak areas of the analyte and the internal standard as a function of analyte concentration with linear regression. The linearity of the method was assessed by performing 5 replicate analyses with 8 different concentrations. For recovery calculations the phosphate buffer saline solution (PBS) was spiked either before or after the extraction with 1 ng/µL of each analyte and the ratios of the peak areas were calculated. The same procedure was conducted with plasma and brain homogenate. The accuracy and precision of the assay were assessed by analyzing blank samples (methanol) spiked with 3 different concentrations in 3 replicates on the same day and on 3 consecutive days for intra- and inter-day precision and accuracy. Precision was calculated as the relative standard deviation (%) and accuracy was determined from the percentage ratio of the measured concentration to the expected concentration.

### Ethics statement

The experimental procedures were conducted in accordance with the ethical principles for animal experimentation adopted by the Brazilian College of Animal Experimentation and were approved by the ethics committee on Animal Care and Use (Comissão de Ética em Cuidados e Uso Animal do Instituto de Química da Universidade de São Paulo – CEUA – IQ-USP) (Permit Number: 15/2011). All surgery was performed under anesthesia, and all efforts were made to minimize animal suffering.

### Biological sample preparation

Plasma and brain samples were obtained from four-month-old Sprague-Dawley rats (n=3). The rats were maintained under a controlled temperature and light-dark cycle with food and water offered *ad libitum*. The animals were anesthetized with an intraperitoneal dose of ketamine hydrochloride (0.9 mL/kg body weight) and xylazine (0.5 mL/kg body weight). Blood was collected from the right atrium of the heart by cardiac puncture into a heparinized tube and centrifuged for 30 min at 4 °C and 1500 x g to separate the plasma. For the analysis, plasma from 3 rats was combined and stored at -20°C until use. Following the blood collection, the rats were immediately decapitated, their whole brains were rapidly excised, and the cortexes were separated and frozen at -20°C. Similarly, the cortex samples (0.2 - 0.3 g) from 3 rats were combined and were homogenized in ice by using an PowerGen 1000 homogeneizer (Fisher Scientific) for 20 s in 5 vol (1.0 - 1.5 mL) of PBS solution (10 mM, pH 7.4) for 2 min on ice. 

### Lipid extraction

Lipids were extracted from plasma and brain samples after saponification step by the Bligh and Dyer method, with modifications [34]. Ice-cold methanol containing BHT (100 µM) and 5(S)-HETE-d8 (0.25 ng/µL, 500 µL) and KOH (1 M) in methanolic solution (500 µL) were added to the samples (500 µL plasma or brain homogenate). The mixtures were incubated for 30 min in the dark at 37°C. The mixture was cooled on ice, acidified with 60 µL HCl (10 M) and then extracted with chloroform/methanol/water. The sample was mixed with a vortex mixer for 1 min and centrifuged at 1500 x g for 5 min at 4°C. The chloroform/methanol layer was dried with nitrogen, and the residue was resuspended in 100 µL of acetonitrile:methanol:H_2_O (52:18:30, v/v/v). Finally, 10 µL of 12(S)-HETE-d8 (12.5 ng/µL) in the same mixture of solvents was added, and the resulting solution was filtered through 0.22 μm Millex Filter Units before injection into the LC-MS/MS system. The injection volume was 10 µL.

### Statistical analysis

The differences between the concentration levels obtained for each isomer (mean ± S.D.) were determined by one way analysis of variance (ANOVA) followed by the Tukey–Kramer multiple comparison test. A *P* value of 0.05 or less was used as the criterion for statistical significance.

## Results and Discussion

The specific and quantitative analysis of the HpDoHE and HDoHE positional isomers is non-trivial due to the structural similarities and common fragmentation patterns of these molecules. For this reason, there have been few reports describing the analysis of HpDoHE and HDoHE by LC-MS/MS [6,35-38]. To the best of our knowledge, this study is the first analytical report describing the detailed MS/MS characterization and the development of an SRM method for reliably discriminating all twelve positional isomers of both HpDoHE and HDoHE. 

### Chromatographic separation of HpDoHE and HDoHE isomers

The UHPLC chromatographic condition optimized by the automated screening study consisted of a C8 column eluted with a gradient solvent system 0.1% ammonium hydroxide in water (pH 10) and methanol:acetonitrile (18:82, v/v).To confirm the resolution of the analytes using the optimized chromatographic condition, a mixture containing all twelve positional isomers of both HpDoHE and HDoHE was analyzed by UHPLC-MS. Figure 2 shows the chromatographic separation of the standardized condition that separates 10 peaks corresponding to the HpDoHE isomers (numbered sequentially from 1-10) and 11 peaks corresponding to HDoHE (numbered sequentially from 11-21).

### Characterization of HpDoHE and HDoHE isomers by MS/MS analysis

For the MS/MS method development we initially focused on the characterization and optimization of MS/MS parameters to obtain the most selective and sensitive ionization and fragmentation conditions for all 24 positional isomers. The HpDoHE and HDoHE generated abundant deprotonated molecules [M-H]^−^ at *m/z* 359 and 343, respectively, which were selected as precursor ions for collision-induced dissociation (CID) in MS/MS analysis. Additionally, the HpDoHE were easily dehydrated [M-H-H_2_0]^−^ during ionization to yield an intense ion at *m/z* 341. Thus, the MS/MS studies of HpDoHE were performed by selecting both the deprotonated (*m/z* 359) and dehydrated molecules (*m/z* 341). The fragmentation spectra of the HpDoHE and HDoHE isomers are shown in Figures 1 and 2, respectively. The MS/MS spectra obtained by selecting the precursor ions at *m/z* 359 and 341 were similar (data not shown). 

The fragmentation of fatty acid hydro(pero)xide ions by CID-MS/MS yields fragment ions that are common to all or more than one isomer (non-specific fragments) as well as fragments that are specific and indicative of the position of the hydroperoxy or hydroxyl group (specific fragments) [39-41]. Non-specific fragments are usually formed through peripheral cleavages, such as the loss of water, CO_2_ or both, whereas isomer-specific fragments are formed through internal cleavages of the carbon-carbon bond. For the HpDoHE, non-specific fragment ions were observed at *m/z* 341, 315 and 297; for the HDoHE, non-specific fragments were observed at *m/z* 325, 299 and 281. In contrast, specific fragment ions were derived from the α- or β-cleavage of the carbon-carbon bond adjacent to the hydroperoxide/hydroxide group. The α- and β-cleavages can occur at the carboxy or methyl side, giving rise to four possible sites of fragmentation (Scheme S2, Supporting information). Moreover, each of these fragmentation paths yields two fragments; one containing the carboxy segment, and the other the methyl segment. For ease of reading, we named these fragment ions according to the three-letter code proposed by the Serhan group, with some modifications (Scheme S2)[37,41]. The expected theoretical fragments formed through these fragmentation paths were listed for each isomer (Tables S1 and S2, Supporting Information) and were compared with the obtained data.

**Figure 3:**
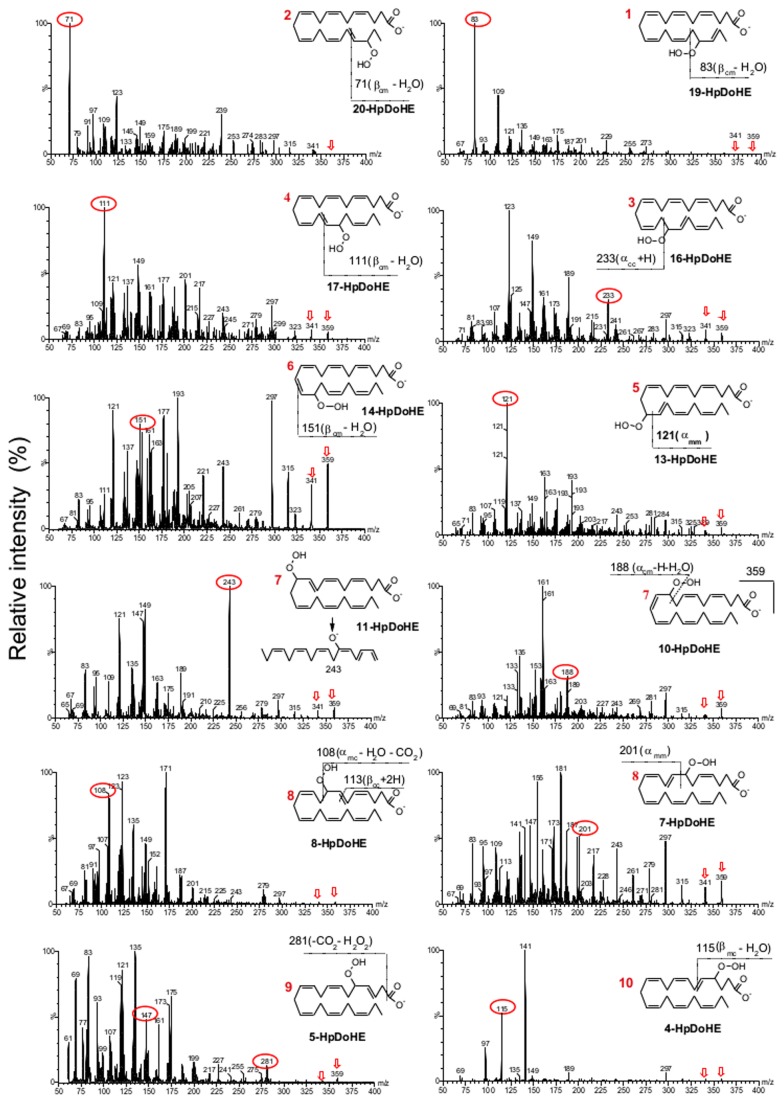
Fragmentation pattern analysis of the HpDoHE isomers – MS/MS spectra obtained from *m/z* 359. The selected fragments for SRM analysis are depicted in each isomer structure. The numbers in red correspond to the peaks observed in the LC-MS analysis (Figure 2).

**Figure 4:**
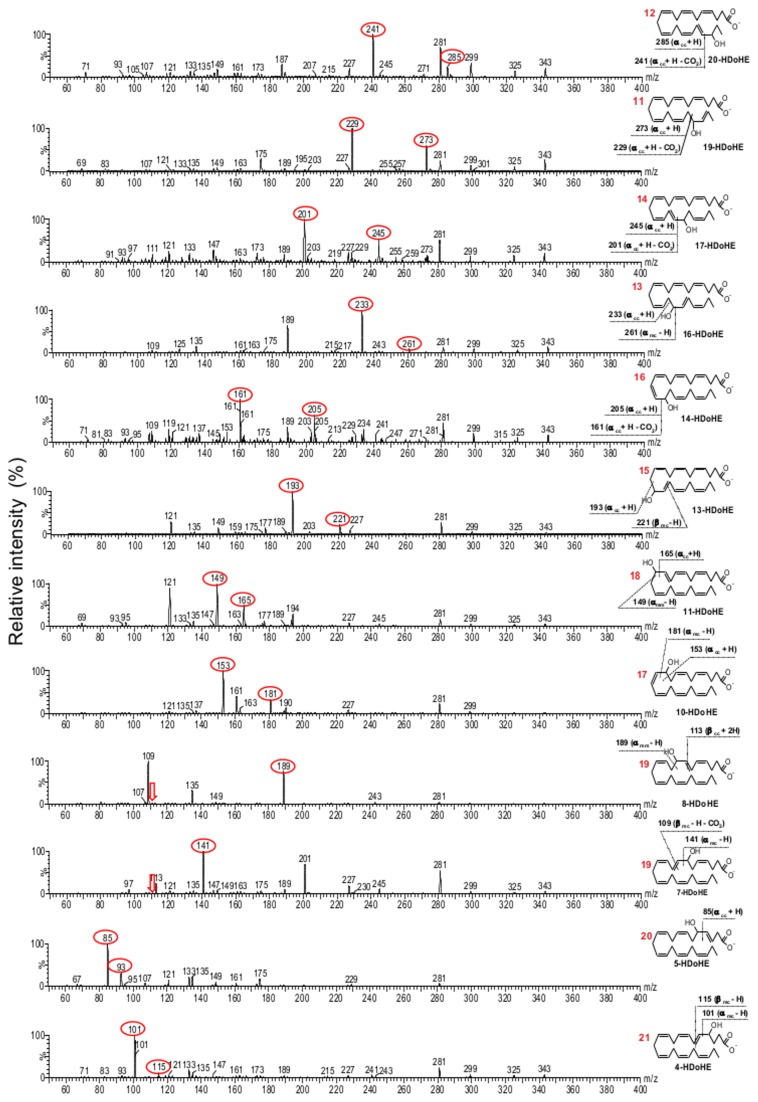
Fragmentation pattern analysis of the HDoHE isomers – MS/MS spectra obtained from *m/z* 343. The selected fragments for SRM analysis are depicted in each isomer structure. The numbers in red correspond to the peaks observed in the LC-MS analysis (Figure 2).

Consistent with previous studies [36,37,39,42], the mono-hydroperoxy (Figure 3) and hydroxy (Figure 4) derivatives of DHA exhibited characteristic fragment ions derived from the α- and β-cleavages followed by one or two hydrogen shifts. MS/MS spectra of the HDoHE isomers showed intense specific fragment ions derived mostly from α-cleavage; in agreement with the data reported by Hong et al.[37] For instance, the 20-, 19-, 17-, 16-, 14-, 13-, 11-, 10- and 5-HDoHE isomers showed intense fragment ions corresponding to the [α_cc_+H]^−^ and [α_cc_+H-CO_2_]^−^ ions, whereas the 8-, 7- and 4-HDoHE isomers showed intense fragment ions corresponding to [α_mc_-H]^−^ and/or [α_mm_]^−^ (Figure 4). In contrast, fragmentation pattern for HpDoHE isomers was more variable. The 20-, 19-, 17-, 16-, 14- and 13-HpDoHE yielded intense ions that corresponded to [α_cc_+H]^−^, [α_cc_+H-CO_2_]^−^ and [β_cm_+H-H_2_O]^−^ and the other isomers (11-, 10-, 8-, 7-, 5- and 4-HpDoHE) exhibited the typical α- and β-cleavages, in addition to other types of chain fragmentations that were not explored further in this study. 

In summary, a comparison of the fragment data obtained in this study and the theoretically expected data allowed us to unambiguously identify and characterize each HpDoHE and HDoHE isomer. 

### Development of the selected reaction monitoring method

**Figure 5:**
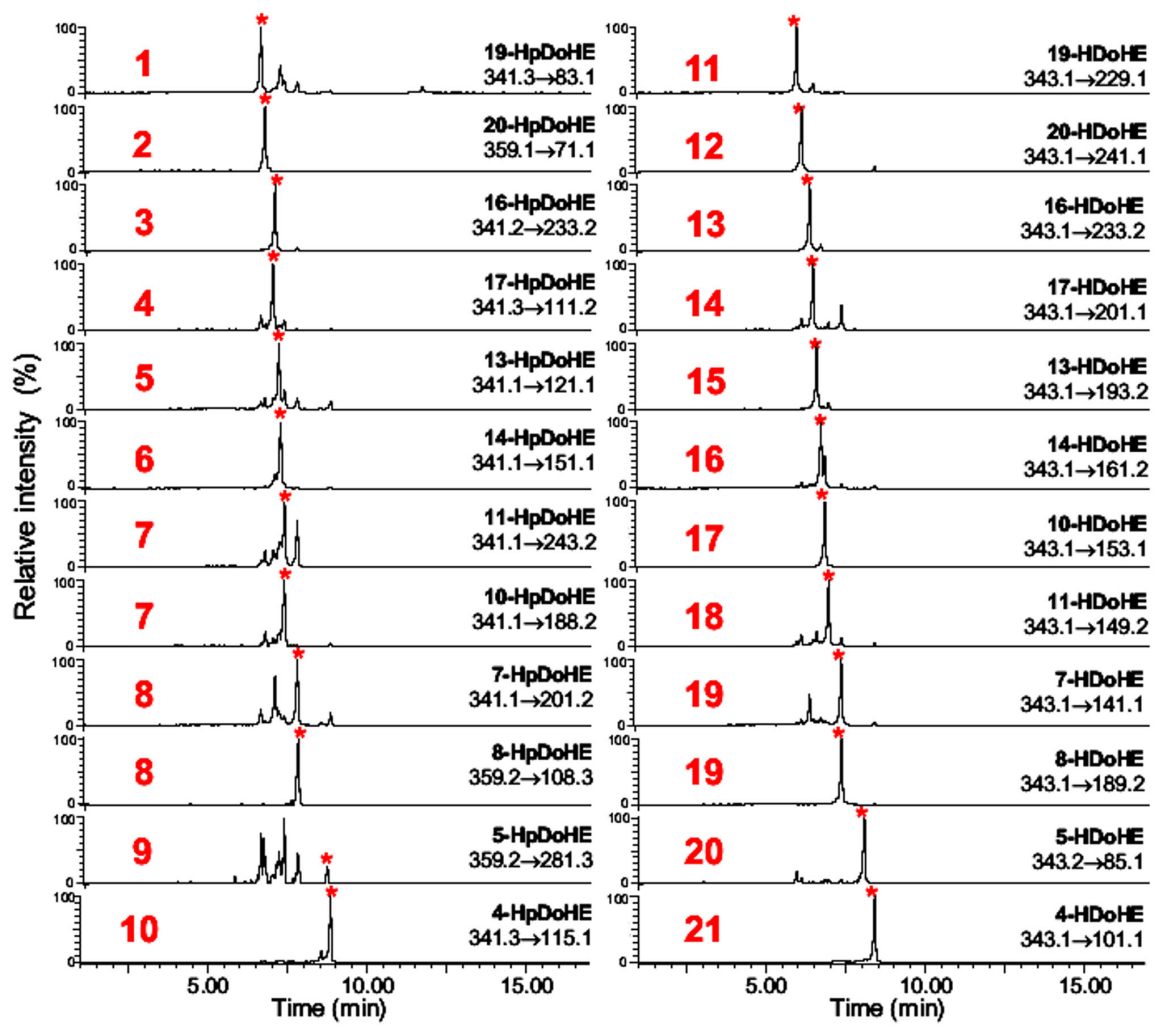
Representative chromatograms of individual SRM transitions selected for each HpDoHE and HDoHE isomer. The numbers in red correspond to the peaks observed in the LC-MS analysis (Figure 2).

Based on the MS/MS spectra of each HpDoHE and HDoHE isomer we developed an SRM method. The selection was based on the intensity and specificity of the fragment ions observed in the MS/MS spectra (Figures 3 and 4).Tables S1 and S2 in the Supporting Information provide the selected fragments used for the SRM method (in red). Figure 5 shows the mass chromatograms obtained using the newly developed SRM method. 

Specific fragment ions selected for HDoHE isomers were all derived from α-cleavage, whereas for HpDoHE isomers, the selected fragment ion was derived mostly from β-cleavage. For some isomers, such as 11-, 7- and 5-HpDoHE, it was necessary to select less specific fragment ions to gain sensitivity in the analysis. As mentioned before, the ESI ionization of hydroperoxides favors the appearance of its corresponding dehydrated ion [39]. Therefore, to gain sensitivity in the analysis, the ion at *m/z* 341 was also selected as an alternative precursor ion for the analysis of the hydroperoxides.

### Quantitative method

The fragment ions selected for the LC-MS/MS-SRM detection of the HpDoHE and HDoHE isomers were chosen to yield the best resolution, selectivity and the highest signal to noise ratio for each isomer. Differently to Yang et al. [43] we did not split the MS method containing all SRM transitions into different acquisition periods. Instead, we chose to split the analysis into two separate methods (one to analyze HpDoHE and the other to analyze the HDoHE isomers) to maintain an adequate number of data points across each chromatographic peak (^≈^ 15 points per peak) and thus increase sensitivity. 

The selected mass transitions and the optimized conditions for the mass spectrometer are presented in Tables 1 and 2. Two mass transitions were selected for each isomer to enhance the detection and quantification selectivity. To determine the limit of detection (LOD) and the linear dynamic ranges for the different isomers, five batches of calibration curves containing 8 different concentrations of each analyte were used. The LOD values were calculated with a signal to noise ratio of 3. The LOD values were within 1−670 pg for HpDoHE and 0.5−8.5 pg for HDoHE injected onto the column, showing that the method is about 10-100 times more sensitive for the detection of the hydroxides compared to hydroperoxides. The batches used in the present work gave linear dynamic ranges of 12.5−1000 pg/µL for HpDoHE and 0.3−100 pg/µL for HDoHE. The *r* ^2^ values determined by regression analysis were 0.99 or greater for each isomer. 

The recoveries for all tested compounds in PBS were good and ranged from 80 to 120 %, with the exception of the 20-HpDoHE which showed a recovery of about 60 % (Table S3 from supporting information). In biological matrices, the average recovery for the HpDoHE isomers was poor (16 ± 22 % in brain and 22 ± 14 % in plasma) compared to the HDoHE isomers (84 ± 20 % in brain and 90 ± 13 % in plasma) which showed good recoveries for all isomers (Table S3 from supporting information). Considering that hydroperoxide loss due to sample processing is around 20 %, we can assume that most of the HpDoHE isomers was either reduced by the antioxidant machinery or degraded by some components of the biological sample. In contrast, recoveries for the hydroxides were good probably reflecting their greater stability in biological samples compared to the hydroperoxides. 

The precision and accuracy of the method showed to be also good. All tested isomers with the exception of 5-HpDoHE and 5-HDoHE present relative standard deviation lower than 8 % and an accuracy higher than 90% (Table S4 and S5 from supporting information).

### Application of the method to biological samples

To demonstrate the applicability of our method as a tool for HpDoHE and HDoHE lipidomic analysis, we used it to detect the basal levels of these isomers in rat plasma and brain samples. All cautions to avoid ex-vivo oxidations were taken, such as keeping samples at low temperatures and using antioxidants and chelating agents during sample preparation and lipid extraction. 

**Figure 6:**
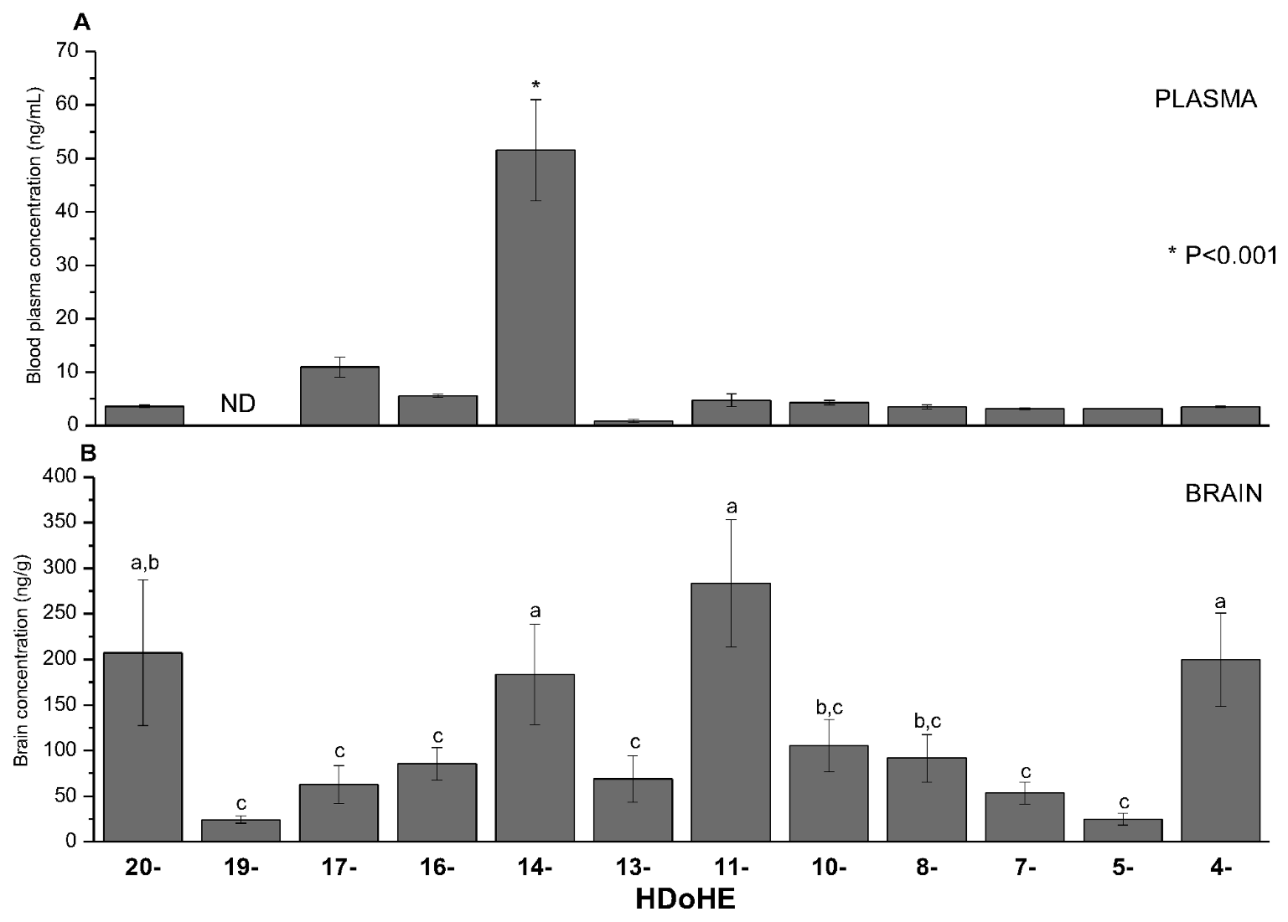
HDoHE profile of rat plasma and brain homogenate samples taken under basal conditions. The results are the mean ± SD of three independent analyses. Values not sharing common superscript are significantly different (*P*<0.05).

 As it would be expected from the recovery studies, the HpDoHE isomers were not detected in the tested biological samples. Among the HDoHE isomers, eleven were detected in the rat plasma. The most abundant isomer observed in plasma was the 14-HDoHE isomer (51.55 ± 9.45 ng/mL), a 12-LOX product, which was present at a 6-10-fold higher concentration than the other isomers (Figure 6A, P<0,001). A similar trend was previously observed by Gomolka et al., who also detected higher levels of 14-HDoHE (65.40 ± 15.84 ng/mL) in whole blood samples from mice [38].

In brain samples, all twelve HDoHE isomers were detected (Figure 6B). To date, the only studies describing the detection of HDoHE in the brain have been performed in models in which the brain homogenates were incubated with DHA or were challenged to produce the oxidized products [6,7,44,45]. To our knowledge, this is the first study describing the detection of the twelve isomers in brain homogenates at basal conditions. 

Interestingly, we found relatively higher levels of HDoHE in the brain than in the plasma samples. This is most likely due to the presence of high concentrations of DHA in nervous tissue. Among the twelve isomers, 20-, 14-, 11- and 4-HDoHE were predominant, and these isomers were all present at similar levels in brain sample (Figure 6B). The 12-LOX is the major LOX in the brain, and this might explain the high levels of 14-HDoHE and 11-HDoHE found in this tissue [46,47]. A predominance of the 20- and 4-HDoHE isomers in brain was also previously reported by Kim et al [45]. These isomers seem to be preferentially accumulated through the non-enzymatic oxidation of DHA [36]. 

Among the less abundant HDoHE isomers, it should be pointed out that we could also detect the 19- and 5-HDoHE isomers, which are known to be specifically formed by singlet oxygen mediated oxidation. Despite the need of further investigations, their detection could serve as a fingerprint for singlet oxygen-mediated oxidation [48,49].

## Conclusions

HpDoHE and HDoHE can act as important lipid mediators in many physiological and pathophysiological events. However, there is little literature describing the quantification of HpDoHE and HDoHE isomers in biological samples. Additionally, the studies that have sought to examine HpDoHE and HDoHE were based on the analysis of lower number of isomers. In this study, we have standardized an LC-MS/MS-SRM method for the analysis of 12 isomers of each HpDoHE and HDoHE. In this way, we are providing a broad and specific method for the analysis of HpDoHE and HDoHE isomers that can be applied to the studies that seek to understand the role of DHA and its oxidation products in biological systems.

## Supporting Information

Method S1 **HpDoHE synthesis by photooxidation.**
(DOCX)

Method S2 **HpDoHE conversion to HDoHE.**
(DOCX)

Scheme S1 **Representative UV spectra of the HpDoHE and HDoHE isomers with (**A**) and without (**B**) conjugated dienes.**
(TIFF)

Scheme S2 **The nomenclature for the HpDoHE and HDoHE chain-cut segments obtained from theoretical MS/MS fragmentation.** This scheme was adapted from Serhan’s group proposed nomenclature for MS/MS ions generated from docosanoids. Each fragment is identified by a 3-letter abbreviation in which: (A) the first Greek letter (α or β) indicates the position of the carbon-carbon bond being cleaved relative to the hydroperoxide/hydroxide group; (B) the second letter indicates whether the cleavage occurred at the carboxy (c) or methyl (m) side; and (C) the third letter indicates the segment that corresponds to the fragment ion, the carboxy (c) or methyl (m) side segment. Thus, with this nomenclature, 4 types of fragment ions can be formed from α-carbon bond cleavage (α_cc_, α_cm_, α_mc_, α_mm_) or from β-carbon bond cleavage (β_cc_, β_cm_, β_mc_, β_mm_). For a more detailed explanation on the fragmentation mechanism, see Murphy et al.[42] and Hong et al.[37].
(TIFF)

Table S1 **Mass of theoretical fragments and *m/z* values of the ions used for the identification of HpDoHE.**
(DOCX)

Table S2 **Mass of theoretical fragments and *m/z* values of the ions used for the identification of HDoHE.**
(DOCX)

Table S3 **Recovery of HpDoHE an HDoHE in different matrix (n=3).**
(DOCX)

Table S4 **Intra-day and inter-day precision (coefficients of variation) and accuracies for the twelve HpDoHE isomers.**
(DOCX)

Table S5 **Intra-day and inter-day precision (coefficients of variation) and accuracies for the twelve HDoHE isomers.**
(DOCX)
