# Supplementary material for: The Development of a Specific and Sensitive LC-MS-Based Method for the Detection and Quantification of Hydroperoxy- and Hydroxydocosahexaenoic Acids as a Tool for Lipidomic Analysis
Source: PLoS One. 2013 Oct 24;8(10):e77561. doi: 10.1371/journal.pone.0077561 (PMC3812029; doi:10.1371/journal.pone.0077561)
Supplement: Method S1 — HpDoHE synthesis by photooxidation. (DOCX) [file pone.0077561.s001.docx]

**Supporting Information**

**Method S1. HpDoHE synthesis by photooxidation.** HpDoHE was synthesized by photooxidation of DHA under an atmosphere saturated with O_2_, and methylene blue was used as the photosensitizer, as previously described.[[1](#_ENREF_1)] Briefly, 100 μL of a methylene blue solution (0.1 M in methanol) were added to 1 g of docosahexaenoic acid dissolved in 50 ml of chloroform. The reaction was conducted in a glass flask (Pyrex, 100 mL) immersed in an ice bath. The mixture was irradiated with two tungsten lamp (luminous intensity of 170 W/m^2^), 10 cm away from the glass flask, for about 2 hours with continuous stirring. The formation of HpDoHE was monitored by thin layer chromatography (TLC) on silica plates eluted with a mixture of chloroform: methanol (90:6, v/v) and by measuring the UV absorbance of the solution at 235 nm. Lipids in TLC plates were visualized by spraying the plates with a 50% sulfuric acid solution followed by heating.

1. Miyamoto S, Martinez GR, Martins APB, Medeiros MHG, Di Mascio P (2003) Direct evidence of singlet molecular oxygen production in the reaction of linoleic acid hydroperoxide with peroxynitrite. Journal of the American Chemical Society 125: 4510-4517.
