# Supplementary material for: The Development of a Specific and Sensitive LC-MS-Based Method for the Detection and Quantification of Hydroperoxy- and Hydroxydocosahexaenoic Acids as a Tool for Lipidomic Analysis
Source: PLoS One. 2013 Oct 24;8(10):e77561. doi: 10.1371/journal.pone.0077561 (PMC3812029; doi:10.1371/journal.pone.0077561)
Supplement: Method S2 — HpDoHE conversion to HDoHE. (DOCX) [file pone.0077561.s002.docx]

**Method S2. HpDoHE conversion to HDoHE**

The conversion of HpDoHE to HDoHE was performed as described by Terao et al.[[1](#_ENREF_1)] To prepare the hydroxide, the HpDoHEs were dried with nitrogen. Then, 1 mL of methanol and 1 mg of sodium borohydride (NaBH_4_) were added. The mixture was incubated on ice for 2 h. Then, 1 mL of H_2_O and 20 μL of HCl (10 M) were added, and the HDoHEs were extracted with 4 mL of hexane:ether (50:50). The organic phase was collected, and the solvent was evaporated with nitrogen gas. Finally, the remaining residue was dissolved in 150 μL of methanol and was stored at -80°C. Absolute concentrations of HDoHE were determined by both UV absorption measurements and HPLC analysis, except for the isomers 5- and 19-HDoHE that were determined only by the second method. Concentration of the isomers having conjugated dienes (ten isomers) were initially determined by UV absorption using ε_235nm_ = 25200 (isomers without conjugated dienes were measured only by HPLC analysis) and then confirmed by HPLC analysis. For determination of hydroxide concentration by HPLC, we injected known concentrations of the corresponding HpDoHE isomer. Concentrations were calculated by measuring the chromatographic areas at 205nm (isomers without conjugated dienes) and 235nm (with conjugated diene) of HpDoHE and HDoHE.

1. Terao J, Shibata SS, Matsushita S (1988) Selective quantification of arachidonic acid hydroperoxides and their hydroxy derivatives in reverse-phase high performance liquid chromatography. Analytical Biochemistry 169: 415-423.
