# Supplementary figures and images for: The Development of a Specific and Sensitive LC-MS-Based Method for the Detection and Quantification of Hydroperoxy- and Hydroxydocosahexaenoic Acids as a Tool for Lipidomic Analysis
Source: PLoS One. 2013 Oct 24;8(10):e77561. doi: 10.1371/journal.pone.0077561 (PMC3812029; doi:10.1371/journal.pone.0077561)

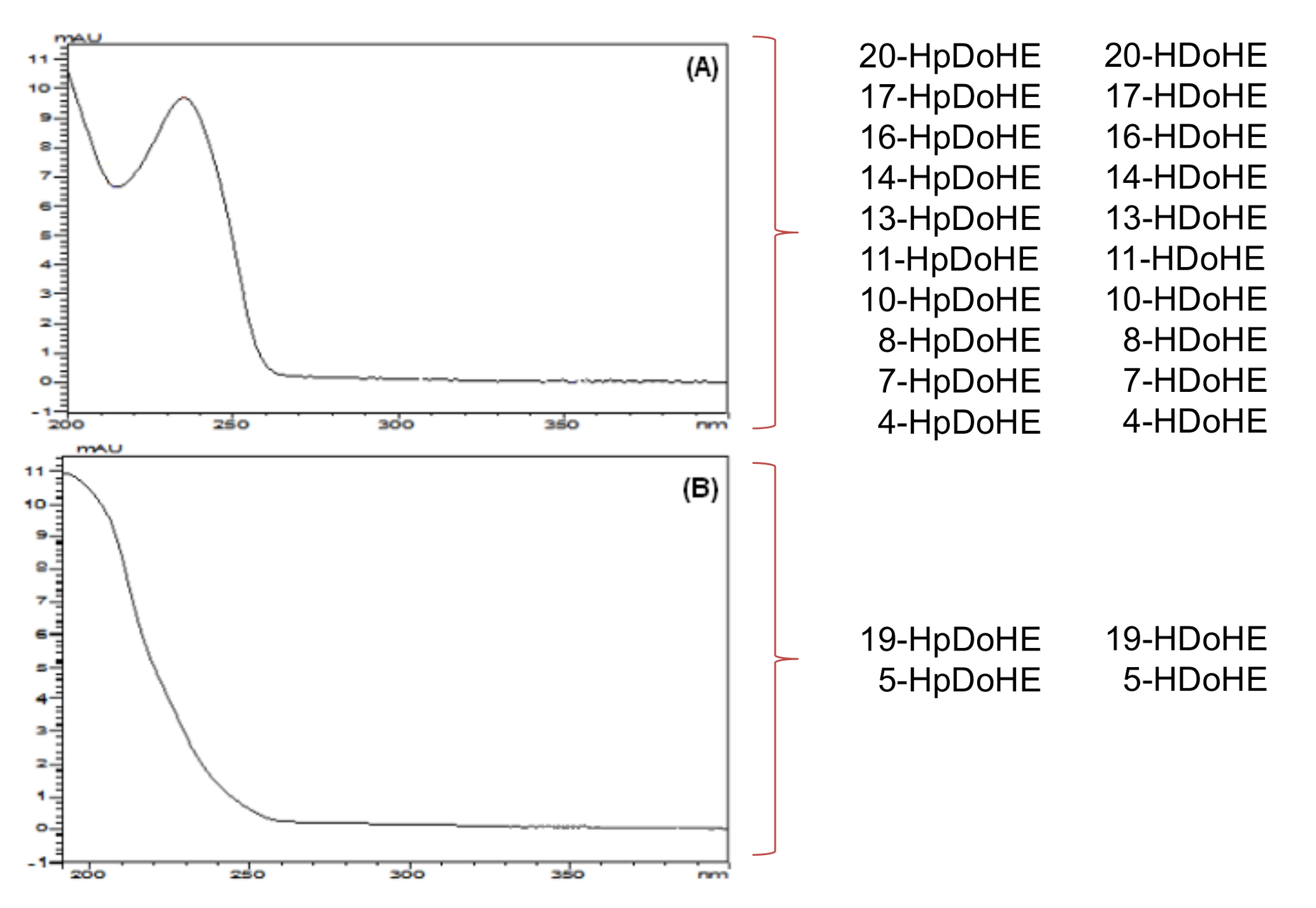

Supplement: Scheme S1 — Representative UV spectra of the HpDoHE and HDoHE isomers with (A) and without (B) conjugated dienes. (TIFF) [file pone.0077561.s003.tiff]

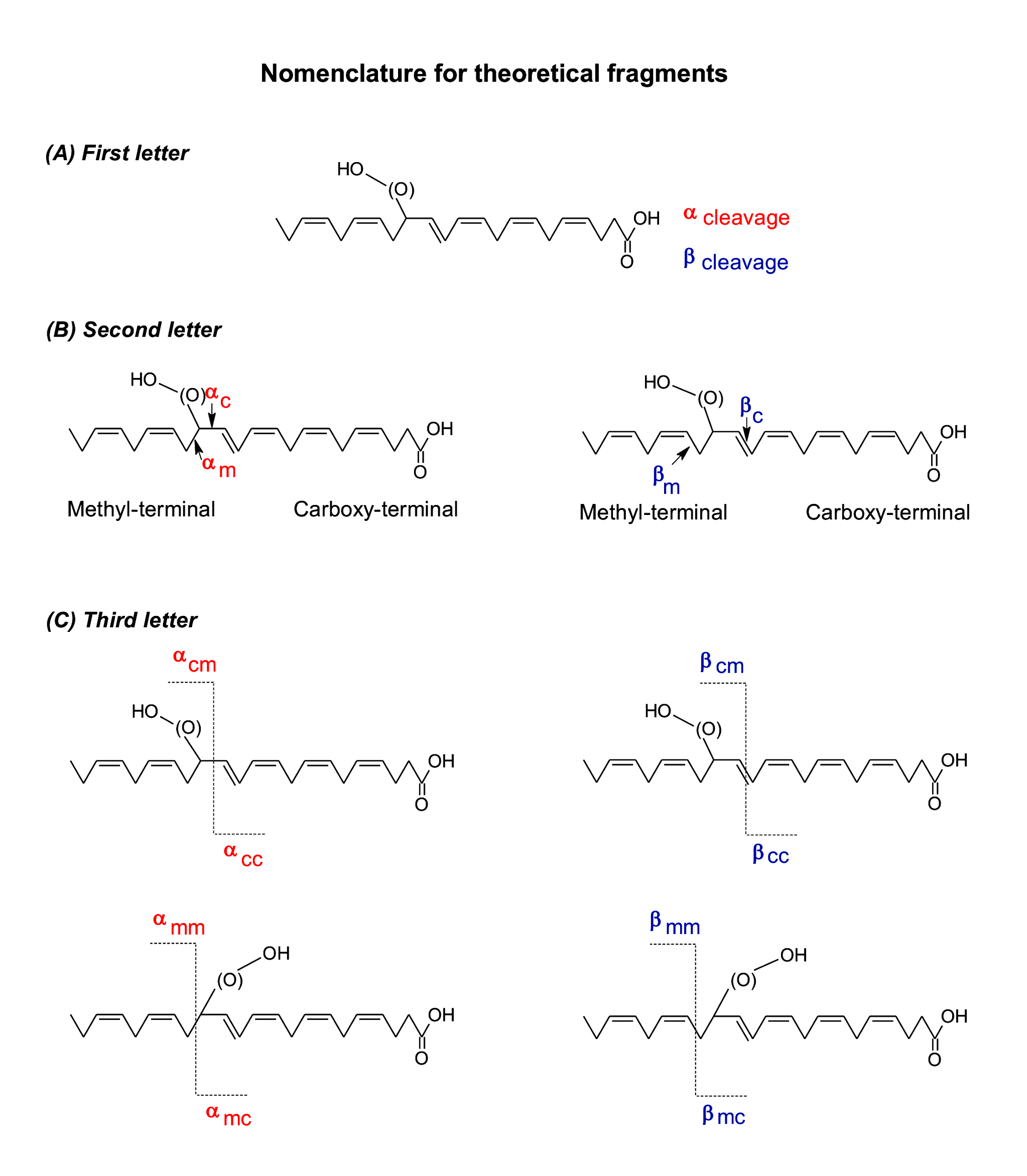

Supplement: Scheme S2 — The nomenclature for the HpDoHE and HDoHE chain-cut segments obtained from theoretical MS/MS fragmentation. This scheme was adapted from Serhan’s group proposed nomenclature for MS/MS ions generated from docosanoids. Each fragment is identified by a 3-letter abbreviation in which: (A) the first Greek letter (α or β) indicates the position of the carbon-carbon bond being cleaved relative to the hydroperoxide/hydroxide group; (B) the second letter indicates whether the cleavage occurred at the carboxy (c) or methyl (m) side; and (C) the third letter indicates the segment that corresponds to the fragment ion, the carboxy (c) or methyl (m) side segment. Thus, with this nomenclature, 4 types of fragment ions can be formed from α-carbon bond cleavage (αcc, αcm, αmc, αmm) or from β-carbon bond cleavage (βcc, βcm, βmc, βmm). For a more detailed explanation on the fragmentation mechanism, see Murphy et al.[42] and Hong et al.[37]. (TIFF) [file pone.0077561.s004.tiff]
