# Supplementary material for: The Development of a Specific and Sensitive LC-MS-Based Method for the Detection and Quantification of Hydroperoxy- and Hydroxydocosahexaenoic Acids as a Tool for Lipidomic Analysis
Source: PLoS One. 2013 Oct 24;8(10):e77561. doi: 10.1371/journal.pone.0077561 (PMC3812029; doi:10.1371/journal.pone.0077561)
Supplement: Table S1 — Mass of theoretical fragments and m/z values of the ions used for the identification of HpDoHE. (DOCX) [file pone.0077561.s005.docx]

**Table S1 Mass of theoreticαl fragments and *m/z* values of the ions used in the identification of HpDoHE**. Ions in bold correspond to the ions selected for SRM method.

| Compound | Loss of | β_cc_ | β_cm_ | α_cc_ | α_cm_ | α_mc_ | α_mm_ | β_mc_ | β_mm_ | Specific chαin-cut ions (m/z) |
| --- | --- | --- | --- | --- | --- | --- | --- | --- | --- | --- |
| 20-HpDoHE |  | 271 | 88 | 284 | 75 | 330 | 29 | 344 | 15 | 285 [αcc+H]or [αmc-H-CO_2_], 239 [αcc-H-CO_2_], 273 [βcc+2H], 229 [βcc+2H-CO_2_], **71 [βcm-H_2_O]** |
|  | -CO_2_ | 227 |  | 240 |  | 286 |  | 300 |  |  |
|  | -CO_2_-H_2_O |  |  |  |  | 268 |  | 282 |  |  |
|  | -H_2_O |  | **71** |  | 57 | 312 |  | 326 |  |  |
| 19-HpDoHE |  | 258 | 101 | 272 | 87 | 318 | 41 | 331 | 28 | 273 [αcc+H]or [αmc-H-CO_2_], 229 [αcc+H-CO_2_], 255 [αmc-H-H_2_O-CO_2_], 215 [βcc+2H-CO_2_], **83 [βcm-H_2_O]** |
|  | -CO2 | 214 |  | 228 |  | 274 |  | 287 |  |  |
|  | -CO_2_-H_2_O |  |  |  |  | **256** |  | 269 |  |  |
|  | -H_2_O |  | **83** |  | 69 | 300 |  | 313 |  |  |
| 17-HpDoHE |  | 231 | 128 | 244 | 115 | 290 | 69 | 304 | 55 | 243 [αcc-H], 201 [αcc+H-CO_2_], 227 [αmc-H-H_2_O-CO_2_], 189 [βcc+2H-CO_2_], **111 [βcm+H-H_2_O]** |
|  | -CO_2_ | 187 |  | 200 |  | 246 |  | 260 |  |  |
|  | -CO_2_-H_2_O |  |  |  |  | 228 |  | 242 |  |  |
|  | -H_2_O |  | **110** |  | 97 | 272 |  | 286 |  |  |
| 16-HpDoHE |  | 218 | 141 | **232** | 127 | 278 | 81 | 291 | 68 | **233 [αcc+H],** 189 [αcc+H-CO_2_], 217 [αmc+H-H_2_O-CO_2_], 189 [βcc+2H-CO_2_], 123 [βcm+H-H_2_O], 107 [αcm-2H-H_2_O] |
|  | -CO_2_ | 174 |  | 188 |  | 234 |  | 247 |  |  |
|  | -CO_2_-H_2_O |  |  |  |  | 216 |  | 229 |  |  |
|  | -H_2_O |  | 123 |  | 109 | 260 |  | 273 |  |  |
| 14-HpDoHE |  | 191 | 168 | 204 | 155 | 250 | 109 | 264 | 95 | 205 [αcc+H], 161 [αcc+H-CO_2_], 189 [αmc+H-H_2_O-CO_2_], 193 [βcc+2H], 149 [βcc+2H-CO_2_], **151 [βcm+H-H_2_O]** |
|  | -CO_2_ | 147 |  | 160 |  | 206 |  | 220 |  |  |
|  | -CO_2_-H_2_O |  |  |  |  | 188 |  | 202 |  |  |
|  | -H_2_O |  | **150** |  | 137 | 232 |  | 246 |  |  |
| 13-HpDoHE |  | 178 | 181 | 192 | 167 | 238 | **121** | 251 | 108 | 193 [αcc+H], 149 [αcc+H-CO_2_], **121 [αmm],** 177 [αmc+H-H2O-CO2], 163 [βcm+H-H_2_O] |
|  | -CO_2_ | 134 |  | 148 |  | 194 |  | 207 |  |  |
|  | -CO_2_-H_2_O |  |  |  |  | 176 |  | 189 |  |  |
|  | -H_2_O |  | 163 |  | 149 | 220 |  | 233 |  |  |
| 11-HpDoHE |  | 151 | 208 | 164 | 195 | 210 | 149 | 224 | 135 | 163 [βmc+H-CO_2_-H_2_O], 121 [αcc+H-CO_2_], 149 [αmm] or [αmc+H-H_2_O-CO_2_], 175 [αcm-2H-H_2_O], 163 [βcm+H-H_2_O], **243*** |
|  | -CO_2_ | 107 |  | 120 |  | 166 |  | 180 |  |  |
|  | -CO_2_-H_2_O |  |  |  |  | 148 |  | 162 |  |  |
|  | -H_2_O |  | 190 |  | 177 | 192 |  | 206 |  |  |
| 10-HpDoHE |  | 138 | 221 | 152 | 207 | 198 | 161 | 211 | 148 | 153 [αcc+H], 135 [αmc+H-H_2_O-CO_2_], 161 [αmm], **188 [αcm-H-H_2_O],** 203 [βcm+H-H_2_O] |
|  | -CO_2_ | 94 |  | 108 |  | 154 |  | 167 |  |  |
|  | -CO_2_-H_2_O |  |  |  |  | 136 |  | 149 |  |  |
|  | -H_2_O |  | 203 |  | **189** | 180 |  | 193 |  |  |
| 8-HpDoHE |  | 111 | 248 | 124 | 235 | 170 | 189 | 184 | 175 | 171 [αmc+H], **108 [αmc-H_2_O-CO_2_],** 81 [αcc+H-H_2_O], 189 [αmm], 123 [βmc+H-H_2_O], 215 [αcm-2H-H_2_O] |
|  | -CO_2_ | 67 |  | 80 |  | 126 |  | 140 |  |  |
|  | -CO_2_-H_2_O |  |  |  |  | **108** |  | 122 |  |  |
|  | -H_2_O |  | 230 |  | 217 | 152 |  | 166 |  |  |
| 7-HpDoHE |  | 98 | 261 | 112 | 249 | 158 | **201** | 171 | 188 | 261 [βcm], 243 [βcm-H_2_O], 171 [βmc], 95 [αmc-H-H_2_O-CO_2_], 68 [αcc+H-CO_2_], **201 [αmm],** 155 [βmc+2H-H_2_O], 109 [βmc-H_2_O-CO_2_], 228 [αcm-2H-H_2_O] |
|  | -CO_2_ | 54 |  | 68 |  | 114 |  | 127 |  |  |
|  | -CO_2_-H_2_O |  |  |  |  | 96 |  | 109 |  |  |
|  | -H_2_O |  | 243 |  | 231 | 140 |  | 153 |  |  |
| 5-HpDoHE |  | 71 | 288 | 84 | 275 | 130 | 229 | 144 | 215 | 123 [αcm-H-H_2_O], 171 [αmc+H], 69 [αmc+H-H_2_O-CO_2_], 227 [αmm-2H], 83 [βmc+H-H_2_O-CO_2_], 257 [αcm-2H-H_2_O], **281 [gcm-2H-H_2_O], 147*** |
|  | -CO_2_ | 27 |  | 40 |  | 86 |  | 100 |  |  |
|  | -CO_2_-H_2_O |  |  |  |  | 68 |  | 82 |  |  |
|  | -H_2_O |  | 270 |  | 257 | 112 |  | 126 |  |  |
| 4-HpDoHE |  | 58 | 301 | 72 | 287 | 118 | 241 | 131 | 229 | 269 [αcm-2H-H_2_O], 229 [βmm], 241 [αmm], **115 [βmc+2H-H_2_O],** 69 [βmc-H_2_O-CO_2_] |
|  | -CO_2_ | 14 |  | 28 |  | 74 |  | 87 |  |  |
|  | -CO_2_-H_2_O |  |  |  |  | 56 |  | 69 |  |  |
|  | -H_2_O |  | 283 |  | 269 | 100 |  | **113** |  |  |
| * unknown fragmentation | | |  |  |  |  |  |  |  |  |
