# Supplementary material for: The Development of a Specific and Sensitive LC-MS-Based Method for the Detection and Quantification of Hydroperoxy- and Hydroxydocosahexaenoic Acids as a Tool for Lipidomic Analysis
Source: PLoS One. 2013 Oct 24;8(10):e77561. doi: 10.1371/journal.pone.0077561 (PMC3812029; doi:10.1371/journal.pone.0077561)
Supplement: Table S2 — Mass of theoretical fragments and m/z values of the ions used for the identification of HDoHE. (DOCX) [file pone.0077561.s006.docx]

**Table S2 Mass of theoretical fragments and *m/z* values of the ions used in the identification of HDoHE.** Ions in bold correspond to the ions selected for SRM method.

| Compound | Loss of | β_cc_ | β_cm_ | α_cc_ | α_cm_ | α_mc_ | α_mm_ | β_mc_ | β_mm_ | Specific chain-cut ions (m/z) |
| --- | --- | --- | --- | --- | --- | --- | --- | --- | --- | --- |
| 20-HDoHE |  | 271 | 72 | **284** | 59 | 314 | 29 | 328 | 15 | **285 [αcc+H],** 271 [αmc+H], **241 [αcc+H-CO_2_]** |
|  | -CO_2_ | 227 |  | **240** |  | 270 |  | 284 |  |  |
|  | -CO_2_-H_2_O |  |  |  |  | 252 |  | 266 |  |  |
|  | -H_2_O |  |  |  | 41 | 296 |  | 310 |  |  |
| 19-HDoHE |  | 258 | 85 | **272** | 71 | 301 | 41 | 315 | 28 | 299 [βmc+2H-CO_2_], **273 [αcc+H]**, 256 [αmc-H-CO_2_], **229 [αcc+H-CO_2_]** |
|  | -CO_2_ | 214 |  | **228** |  | 257 |  | 271 |  |  |
|  | -CO_2_-H_2_O |  |  |  |  | 239 |  | 253 |  |  |
|  | -H_2_O |  |  |  | 53 | 283 |  | 297 |  |  |
| 17-HDoHE |  | 231 | 112 | **244** | 99 | 274 | 69 | 288 | 55 | 273[αmc-H], **245 [αcc+H] or [βmc+H-CO_2_]** , 227 [αmc-H-H_2_O-CO2**], 201 [αcc+H-CO_2_],** 189 [βcc+2H-CO_2_] |
|  | -CO_2_ | 187 |  | **200** |  | 230 |  | 244 |  |  |
|  | -CO_2_-H_2_O |  |  |  |  | 212 |  | 226 |  |  |
|  | -H_2_O |  | 94 |  | 81 | 256 |  | 270 |  |  |
| 16-HDoHE |  | 218 | 125 | **232** | 111 | **262** | 81 | 275 | 68 | **261 [αmc-H], 233[αcc+H] or [βmc+2H-CO2],** 215 [βmc+2H-H_2_O-CO_2_], 189 [αcc+H-CO2], 125 [βcm] |
|  | -CO_2_ | 174 |  | 188 |  | 218 |  | 231 |  |  |
|  | -CO_2_-H_2_O |  |  |  |  | 200 |  | 213 |  |  |
|  | -H_2_O |  | 107 |  | 93 | 244 |  | 257 |  |  |
| 14-HDoHE |  | 191 | 153 | **204** | 139 | 234 | 109 | 248 | 95 | **205 [αcc+H],** 189 [αmc-H-H_2_O-CO2], **161 [αcc+H-CO_2_],** 153 [βcm], 149 [βcc+2H-CO_2_] |
|  | -CO_2_ | 147 |  | **160** |  | 190 |  | 204 |  |  |
|  | -CO_2_-H_2_O |  |  |  |  | 172 |  | 186 |  |  |
|  | -H_2_O |  | 135 |  | 121 | 216 |  | 230 |  |  |
| 13-HDoHE |  | 178 | 165 | **192** | 151 | 222 | **121** | 235 | 108 | 221 [αmc-H], **193 [αcc+H] or [βmc+2H-CO_2_],** 177 [αmc-H-H_2_O-CO_2_], 149 [αcc+H-CO_2_], 135 [βcm+H-H_2_O], **121 [αmm]** |
|  | -CO_2_ | 134 |  | 148 |  | 178 |  | 191 |  |  |
|  | -CO_2_-H_2_O |  |  |  |  | 160 |  | 173 |  |  |
|  | -H_2_O |  | 147 |  | 133 | 204 |  | 217 |  |  |
| 11-HDoHE |  | 151 | 192 | **164** | 179 | 194 | **149** | 208 | 135 | 177 [αcm+H-H2O], **165 [αcc+H] or [βmc+H-CO2], 149 [βcc-2H] or [αmm],** 121 [βcc+H-CO_2_], 163 [βcm-H-H_2_O] |
|  | -CO_2_ | 107 |  | 120 |  | 150 |  | 164 |  |  |
|  | -CO_2_-H_2_O |  |  |  |  | 132 |  | 146 |  |  |
|  | -H_2_O |  | 174 |  | 161 | 176 |  | 190 |  |  |
| 10-HDoHE |  | 138 | 205 | **152** | 191 | **182** | 161 | 195 | 148 | 188 [βcm+H-H_2_O], **181[αmc-H],** 161[αmm], **153 [αcc+H] or [βmc+2H-CO_2_],** 135 [βmc+2H-H_2_O-CO_2_] |
|  | -CO_2_ | 94 |  | 108 |  | 138 |  | 151 |  |  |
|  | -CO_2_-H_2_O |  |  |  |  | 120 |  | 133 |  |  |
|  | -H_2_O |  | 187 |  | 173 | 164 |  | 177 |  |  |
| 8-HDoHE |  | **111** | 232 | 124 | 219 | 154 | **189** | 168 | 175 | **189 [αmm],** 149 [βmc-H-H_2_O], 135 [αmc-H-H_2_O], **113 [βcc+2H],** 109 [αcc-H-H_2_O] |
|  | -CO_2_ | 67 |  | 80 |  | 110 |  | 124 |  |  |
|  | -CO_2_-H_2_O |  |  |  |  | 92 |  | 106 |  |  |
|  | -H_2_O |  | 214 |  | 201 | 136 |  | 150 |  |  |
| 7-HDoHE |  | 97 | 245 | 112 | 231 | **142** | 201 | 155 | 188 | 245 [βcm], 227 [βcm-H_2_O], 201 [αmm], **141 [αmc-H], 109 [βmc-2H-CO_2_],** 97[αmc-H-CO_2_] |
|  | -CO_2_ | 53 |  | 68 |  | 98 |  | **111** |  |  |
|  | -CO_2_-H_2_O |  |  |  |  | 80 |  | 93 |  |  |
|  | -H_2_O |  | 227 |  | 213 | 124 |  | 137 |  |  |
| 5-HDoHE |  | 71 | 273 | **84** | 259 | 114 | 229 | 128 | 215 | 229 [αmm], 95 [αmc-H-H_2_O] **,93*, 85 [αcc+H]** or [βmc+H-CO_2_] |
|  | -CO_2_ |  |  |  |  | 70 |  | **84** |  |  |
|  | -CO_2_-H_2_O |  |  |  |  | 52 |  | 66 |  |  |
|  | -H_2_O |  | 255 |  | 241 | 96 |  | 110 |  |  |
| 4-HDoHE |  | 58 | 285 | 72 | 271 | **102** | 241 | **115** | 228 | **115 [βmc], 101[αmc-H],** 83 [αmc-H-H_2_O], 71[αcc-H] or [βcc-H_2_O] |
|  | -CO_2_ | 14 |  | 28 |  | 58 |  | 71 |  |  |
|  | -CO_2_-H_2_O |  |  |  |  | 40 |  | 53 |  |  |
|  | -H_2_O |  | 267 |  | 253 | 84 |  | 97 |  |  |
| * unknown fragmentation | | |  |  |  |  |  |  |  |  |
