# Supplementary material for: The Development of a Specific and Sensitive LC-MS-Based Method for the Detection and Quantification of Hydroperoxy- and Hydroxydocosahexaenoic Acids as a Tool for Lipidomic Analysis
Source: PLoS One. 2013 Oct 24;8(10):e77561. doi: 10.1371/journal.pone.0077561 (PMC3812029; doi:10.1371/journal.pone.0077561)
Supplement: Table S3 — Recovery of HpDoHE an HDoHE in different matrix (n=3). (DOCX) [file pone.0077561.s007.docx]

**Table S3. Recovery of HpDoHE an HDoHE in different matrices (n=3)**.

|  |  | | Recovery (%) | | | |
| --- | --- | --- | --- | --- | --- | --- |
|  | | Isomer | | PBS | Brain | Plasma |
| HpDoHE | | 20 | | 60.9 | nd | nd |
|  |  | 19 | | 123.0 | 33.4 | 31.3 |
|  |  | 17 | | 91.4 | 2.1 | 11.3 |
|  |  | 16 | | 115.0 | 18.6 | 33.7 |
|  |  | 14 | | 83.8 | 2.5 | 15.6 |
|  |  | 13 | | 83.4 | 6.3 | 17.9 |
|  |  | 11 | | 94.5 | 3.0 | 10.9 |
|  |  | 10 | | 80.2 | 69.5 | 40.3 |
|  |  | 8 | | 112.7 | nd | 43.4 |
|  |  | 7 | | 85.6 | 3.3 | 32.5 |
|  |  | 5 | | 90.4 | 19.3 | 1.9 |
|  |  | 4 | | 84.5 | 1.9 | 8.3 |
| HDoHE | | 20 | | 84.7 | 45.9 | 96.6 |
|  |  | 19 | | 99.5 | 60.5 | 120.1 |
|  |  | 17 | | 88.8 | 78.2 | 90.2 |
|  |  | 16 | | 82.8 | 67.5 | 80.4 |
|  |  | 14 | | 111.6 | 92.4 | 107.9 |
|  |  | 13 | | 88.4 | 125.2 | 87.8 |
|  |  | 11 | | 89.3 | 85.2 | 86.0 |
|  |  | 10 | | 87.7 | 89.3 | 90.1 |
|  |  | 8 | | 89.3 | 86.5 | 86.7 |
|  |  | 7 | | 89.7 | 82.1 | 79.7 |
|  |  | 5 | | 101.0 | 101.1 | 80.9 |
|  |  | 4 | | 95.8 | 89.8 | 72.2 |

For recovery experiments each sample was spiked with 1 ng/µl of each isomer. Abbreviation: nd means not detected
