# Supplementary material for: The Development of a Specific and Sensitive LC-MS-Based Method for the Detection and Quantification of Hydroperoxy- and Hydroxydocosahexaenoic Acids as a Tool for Lipidomic Analysis
Source: PLoS One. 2013 Oct 24;8(10):e77561. doi: 10.1371/journal.pone.0077561 (PMC3812029; doi:10.1371/journal.pone.0077561)
Supplement: Table S4 — Intra-day and inter-day precision (coefficients of variation) and accuracies for the twelve HpDoHE isomers. (DOCX) [file pone.0077561.s008.docx]

**Table S4 Intra-day and inter-day precision (coefficients of variation) and accuracies for the twelve HpDoHE isomers.**

| Isomer | | Concentration (ng/μl) | | Intra-day (n=3) | | | |  | | Inter-day (n=3) | | | |  |
| --- | --- | --- | --- | --- | --- | --- | --- | --- | --- | --- | --- | --- | --- | --- |
|  |  |  |  | Precision (%) | | Accuracy (%) | |  |  | Precision (%) | | Accuracy (%) | |  |
| HpDoHE | 20 | | 2 | | 6.7 | | 105.0 | |  | | 11.5 | | 114.2 | |
|  |  |  | 4 | | 10.8 | | 103.3 | |  | | 16.9 | | 109.6 | |
|  | 19 | | 2 | | 5.7 | | 101.1 | |  | | 20.4 | | 124.7 | |
|  |  |  | 4 | | 9.3 | | 101.0 | |  | | 8.6 | | 114.8 | |
|  | 17 | | 2 | | 15.9 | | 94.8 | |  | | 24.4 | | 116.1 | |
|  |  |  | 4 | | 8.0 | | 98.2 | |  | | 15.0 | | 104.7 | |
|  | 16 | | 2 | | 11.2 | | 102.2 | |  | | 17.7 | | 123.8 | |
|  |  |  | 4 | | 10.0 | | 100.4 | |  | | 15.1 | | 109.8 | |
|  | 14 | | 2 | | 16.9 | | 101.6 | |  | | 22.1 | | 129.2 | |
|  |  |  | 4 | | 7.9 | | 100.7 | |  | | 15.8 | | 114.8 | |
|  | 13 | | 2 | | 14.5 | | 104.0 | |  | | 18.4 | | 118.2 | |
|  |  |  | 4 | | 9.8 | | 99.1 | |  | | 13.8 | | 103.5 | |
|  | 11 | | 2 | | 24.3 | | 103.1 | |  | | 27.2 | | 97.1 | |
|  |  |  | 4 | | 11.2 | | 102.8 | |  | | 19.0 | | 121.6 | |
|  | 10 | | 2 | | 7.5 | | 104.9 | |  | | 14.7 | | 119.6 | |
|  |  |  | 4 | | 11.8 | | 100.2 | |  | | 13.5 | | 106.8 | |
|  | 8 | | 2 | | 7.2 | | 95.8 | |  | | 15.6 | | 110.3 | |
|  |  |  | 4 | | 11.5 | | 98.6 | |  | | 13.0 | | 108.6 | |
|  | 7 | | 2 | | 10.3 | | 97.4 | |  | | 26.9 | | 128.5 | |
|  |  |  | 4 | | 8.6 | | 100.4 | |  | | 13.3 | | 108.2 | |
|  | 5 | | 2 | | 13.3 | | 86.9 | |  | | 17.0 | | 46.5 | |
|  |  |  | 4 | | 36.8 | | 174.9 | |  | | 8.4 | | 132.0 | |
|  | 4 | | 2 | | 11.7 | | 103.7 | |  | | 25.6 | | 134.5 | |
|  |  |  | 4 | | 9.3 | | 99.7 | |  | | 14.3 | | 113.5 | |
