# Supplementary material for: The Development of a Specific and Sensitive LC-MS-Based Method for the Detection and Quantification of Hydroperoxy- and Hydroxydocosahexaenoic Acids as a Tool for Lipidomic Analysis
Source: PLoS One. 2013 Oct 24;8(10):e77561. doi: 10.1371/journal.pone.0077561 (PMC3812029; doi:10.1371/journal.pone.0077561)
Supplement: Table S5 — Intra-day and inter-day precision (coefficients of variation) and accuracies for the twelve HDoHE isomers. (DOCX) [file pone.0077561.s009.docx]

**Table S5 Intra-day and inter-day precision (coefficients of variation) and accuracies for the twelve HDoHE isomers.**

| Isomer | | Concentration (ng/μl) | | Intra-day (n=3) | | | |  | | Inter-day (n=3) | | |  |
| --- | --- | --- | --- | --- | --- | --- | --- | --- | --- | --- | --- | --- | --- |
|  |  |  |  | Precision (%) | | Accuracy (%) | |  |  | Precision (%) | Accuracy (%) | |  |
| HDoHE | 20 | | 2 | | 5.0 | | 97.8 | |  | 2.7 | | 101.4 | |
|  |  | | 4 | | 5.6 | | 100.7 | |  | 1.9 | | 99.1 | |
|  | 19 | | 2 | | 5.9 | | 98.0 | |  | 2.7 | | 103.1 | |
|  |  | | 4 | | 7.0 | | 101.2 | |  | 2.1 | | 99.2 | |
|  | 17 | | 2 | | 5.9 | | 93.0 | |  | 4.9 | | 100.9 | |
|  |  | | 4 | | 4.3 | | 100.3 | |  | 4.4 | | 104.7 | |
|  | 16 | | 2 | | 6.0 | | 95.2 | |  | 4.1 | | 104.4 | |
|  |  | | 4 | | 5.3 | | 100.5 | |  | 2.0 | | 99.7 | |
|  | 14 | | 2 | | 5.0 | | 95.2 | |  | 6.0 | | 105.4 | |
|  |  | | 4 | | 4.4 | | 100.3 | |  | 2.2 | | 100.7 | |
|  | 13 | | 2 | | 5.9 | | 94.6 | |  | 4.8 | | 105.3 | |
|  |  | | 4 | | 5.8 | | 100.3 | |  | 2.9 | | 101.0 | |
|  | 11 | | 2 | | 3.2 | | 96.9 | |  | 5.4 | | 104.9 | |
|  |  | | 4 | | 5.3 | | 101.1 | |  | 4.6 | | 98.9 | |
|  | 10 | | 2 | | 2.5 | | 98.0 | |  | 5.1 | | 104.3 | |
|  |  | | 4 | | 6.2 | | 98.3 | |  | 2.3 | | 99.1 | |
|  | 8 | | 2 | | 6.2 | | 93.0 | |  | 3.5 | | 103.1 | |
|  |  | | 4 | | 5.1 | | 99.6 | |  | 2.1 | | 98.4 | |
|  | 7 | | 2 | | 6.7 | | 93.0 | |  | 6.1 | | 107.0 | |
|  |  | | 4 | | 6.1 | | 98.4 | |  | 1.6 | | 99.9 | |
|  | 5 | | 2 | | 13.2 | | 101.1 | |  | 34.4 | | 66.1 | |
|  |  | | 4 | | 4.5 | | 103.6 | |  | 2.5 | | 97.6 | |
|  | 4 | | 2 | | 7.7 | | 92.7 | |  | 9.7 | | 109.6 | |
|  |  | | 4 | | 5.0 | | 101.3 | |  | 4.8 | | 103.6 | |
